# Chemotherapy plus Erlotinib versus Chemotherapy Alone for Treating Advanced Non-Small Cell Lung Cancer: A Meta-Analysis

**DOI:** 10.1371/journal.pone.0131278

**Published:** 2015-07-06

**Authors:** J. L. Xu, B. Jin, Z. H. Ren, Y. Q. Lou, Z. R. Zhou, Q. Z. Yang, B. H. Han

**Affiliations:** 1 Department of Pulmonary, Shanghai Chest Hospital, Shanghai Jiaotong University, Shanghai, China; 2 The Ninth People's Hospital of Shanghai, Shanghai Jiaotong University, Shanghai, China; 3 Department of Oncology, Fudan University Shanghai Cancer Center, Shanghai Medical College, Shanghai, China; 4 Department of Gynecology, Women and Child Care of Heyuan, Guangdong, China; Shanghai Jiao Tong University School of Medicine, CHINA

## Abstract

**Background:**

Whether a combination of chemotherapy and erlotinib is beneficial for advanced non-small cell lung cancer (NSCLC) remains controversial. This study aimed to summarize the currently available evidence and compare the efficacy and safety of chemotherapy plus erlotinib versus chemotherapy alone for treating advanced NSCLC.

**Methods:**

EMBASE, PubMed, and the Cochrane Central Register of Controlled Trials were searched for relevant studies. Our protocol was registered in PROSPERO (CRD42014015015).

**Results:**

Nine randomized controlled trials with a total of 3599 patients were included. Compared to chemotherapy alone, chemotherapy plus erlotinib was superior in PFS (HR = 0.76 [95% CI 0.62, 0.92], P = 0.006), and no statistically significant difference was observed in OS (HR = 0.94 [95% CI 0.86, 1.03], P = 0.16). Intercalated erlotinib plus chemotherapy demonstrated improvements in PFS (HR = 0.67 [95% CI 0.50, 0.91], P = 0.009) and OS (HR = 0.82 [95% CI 0.69, 0.98], P = 0.03). Continuous erlotinib plus chemotherapy treatment failed to demonstrate improvements in PFS (HR = 0.91 [95% CI 0.80, 1.04], P = 0.16) and OS (HR = 0.98 [95% CI 0.89, 1.09], P = 0.75). The association of chemotherapy plus erlotinib with improvement in PFS was significant in never smoking patients (HR = 0.46 [95% CI 0.37, 0.56], P<0.00001) but not in smoking patients (HR = 0.70 [95% CI 0.49, 1.00], P = 0.05). Among patients with EGFR mutant tumors, chemotherapy plus erlotinib demonstrated significant improvements in PFS (HR = 0.31 [95% CI 0.17, 0.58], P = 0.0002) and OS (HR = 0.52 [95% CI 0.30, 0.88], P = 0.01). Among patients with EGFR wild-type tumors, no statistically significant difference was observed with respect to PFS (HR = 0.87 [95% CI 0.70, 1.08], P = 0.21) and OS (HR = 0.78 [95% CI 0.59, 1.01], P = 0.06).

**Conclusion:**

Combination of chemotherapy and erlotinib is a viable treatment option for patients with NSCLC, especially for patients who never smoked and patients with EGFR mutation-positive disease. In addition, intercalated administration is an effective combinatorial strategy.

## Introduction

Lung cancer is the most common cause of cancer-related deaths worldwide [1], and approximately 85 to 90% of all lung cancer cases are non-small cell lung cancer (NSCLC). More than 70% of lung cancer cases are diagnosed at an advanced stage, which means that surgery is not an option for most of these patients [1, 2]. Furthermore, chemotherapy alone fails to provide patients with significant effects in overall survival [3, 4]. Drug resistance is one of the major reasons why patients fail to benefit from chemotherapy as most patients’ tumors quickly develop acquired resistance to chemotherapeutic drugs [5, 6].

Erlotinib is an oral epidermal growth factor receptor tyrosine kinase inhibitor (EGFR-TKI), and has been considered as the standard treatment for patients with EGFR mutant tumors. It was demonstrated that EGFR-TKIs could prolong overall survival in EGFR-unselected patients with NSCLC [7]. In the last decade, several studies evaluated EGFR-TKIs in combination with standard chemotherapy for patients with advanced NSCLC [8–17]. Continuous erlotinib in combination with carboplatin based chemotherapy failed to demonstrate a survival advantage over carboplatin based chemotherapy alone in patients with previously untreated advanced NSCLC [16]. The GFPC 10.02 study also showed that intercalated erlotinib in combination with docetaxel was not more effective than docetaxel alone as a second-line treatment for advanced NSCLC with wild-type or unknown EGFR status [10]. Some experts believe that EGFR-TKIs cause a G1 cell-cycle arrest, which can inhibit the cell-cycle-dependent cytotoxic effects of chemotherapy [18]. However, a multicenter phase II trial showed the superior efficacy of the combination of pemetrexed and erlotinib over pemetrexed alone [13]. FASTACT-2 [11], a phase III study, also showed significant improvement in efficacy with an intercalated regimen of chemotherapy and an EGFR-TKI for patients with advanced NSCLC. Whether the combination of chemotherapy and erlotinib is beneficial for advanced NSCLC remains controversial. It is also unclear which population of patients may gain the greatest benefit from this combinational approach. Therefore, we performed this meta-analysis of randomized clinical trials to assess the efficacy and safety of erlotinib in combination with standard chemotherapy versus chemotherapy alone for the treatment of patients with advanced NSCLC and explore whether the outcomes vary by different patient subgroups.

## Materials and Methods

The methods are based on our previously described protocol [19]. We conducted this meta-analysis with the guidance of the Preferred Reporting Items for Systematic Reviews and Meta-analyses (PRISMA) Statement [20].

### Search strategy

Two authors (Ren ZH, Xu JL) independently carried out a comprehensive systematic search for published articles from inception to October 22, 2014 using the PubMed, EMBASE, and Cochrane databases. Moreover, we searched the ClinicalTrials.gov website for information about registered randomized controlled clinical trials (RCTs). The search was limited to articles published in English. We resolved any disagreements through discussion with a third person (Han BH). The following search items were used: ("Randomized Controlled Trials as Topic"[Mesh] OR Randomized Controlled Trial [Publication Type]) OR random*) AND ("Lung Neoplasms"[Mesh] OR nsclc OR non-small cell OR lung neoplasm* OR lung tumor* OR lung carcinoma* OR lung cancer*) AND ("Tarceva” OR erlotinib [Title/Abstract] OR "erlotinib" [Supplementary Concept]). The references from the included studies and previous meta-analyses were also manually examined.

### Eligibility criteria

The inclusion criteria were as follows: 1. Randomized controlled clinical trials; 2. Studies comparing erlotinib plus standard chemotherapy to standard chemotherapy alone; 3. At least one of the two endpoints (PFS, OS) was reported. The exclusion criteria were as follows: 1. Single arm studies; 2. Observational cohort studies; 3. Erlotinib was given after chemotherapy was completed or chemotherapy was given after erlotinib was discontinued. 4. Maintenance therapy studies. When duplicate publications were identified, the most complete reports were included.

### Quality assessment

Two authors (Zhou ZR, Xu JL) independently assessed the quality of the trials using the criteria outlined in the Cochrane Handbook for Systematic Reviews of Interventions, which appraised sequence generation, allocation concealment, performance bias, detection bias, attrition bias, reporting bias, and other biases. Disagreements between reviewers were resolved by discussion with a third person (Jin B).

### Data collection

Two authors (Yang QZ, Xu JL) independently extracted data about the first author, year of publication, treatment comparison, drug delivery, regimens of each arm, the number of patients enrolled, age, hazard ratios (HR) and 95% confidence intervals (CI) for progression free survival (PFS) and overall survival (OS), median PFS and median OS, and adverse events (AEs). If HRs were not directly reported, we contacted the authors of the primary studies for additional data. If we were unable to contact the authors, we extracted data from survival curves [21].

### Statistical analysis and publication bias

Two authors (Lou YQ, Xu JL) performed the statistical analyses. A fixed-effect meta-analysis was used to calculate pooled HRs for PFS and OS, and OR for AEs, together with 95% CIs. We assessed the presence of statistical heterogeneity among the studies by using the Q statistic, and the magnitude of heterogeneity was assessed using the *I* ^*2*^ statistic. If statistical heterogeneity was detected, in which P<0.10 or *I* ^*2*^ was more than 50%, a random-effect meta-analysis was used. It was explored by subgroup analysis as follows: smoking or not, ethnicity, intercalated therapy (receiving chemotherapy with intercalated erlotinib) or continuous therapy (receiving chemotherapy with continuous erlotinib), EGFR-mutant or EGFR wild-type. The meta-analysis results were displayed as forest plots. All calculations were performed using the Review Manager 5.3. Publication bias was assessed by the construction of funnel plots.

## Results

### Eligible studies

**Fig 1:**
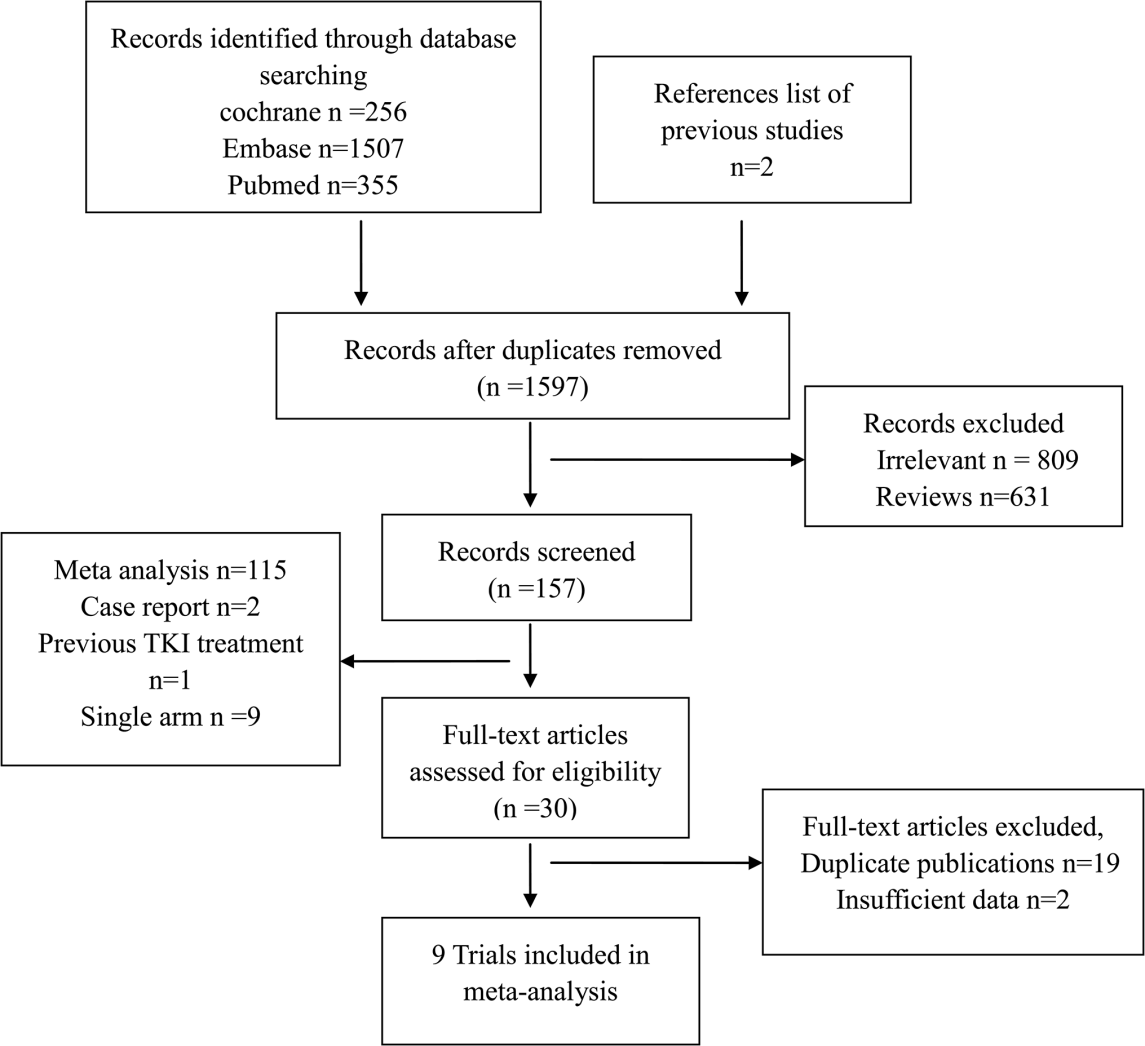
Flow Diagram of Included and Excluded Studies.

A total of 1597 articles were identified by the initial search strategy. After excluding irrelevant studies, review articles, RCTs without adequate data, meta-analyses, observational studies, single arm trials and case reports, nine trials were finally included for this meta-analysis (S1 File shows a list of full-text excluded articles). The selection steps are summarized in the flow chart shown in Fig 1. These trials enrolled a total of 3599 patients. Among them [8–16], four trials were placebo-controlled double-blinded trials [11, 14–16]. Five trials used intercalated erlotinib plus chemotherapy as the drug delivery method, while other four trials used continuous erlotinib plus chemotherapy treatment. Three trials enrolled an Asian-dominant population [11, 13, 14]http://www.sciencedirect.com/science/article/pii/S1525730411002105 - bib22, while other six trials enrolled a Caucasian-dominant population [8–10, 12, 15, 16]. One trial only enrolled patients who never smoked [13]. Five studies compared gemcitabine based chemotherapy plus erlotinib versus gemcitabine based chemotherapy alone [8, 11, 12, 14, 15], two trials compared pemetrexed and erlotinib with either pemetrexed or erlotinib alone [9, 13], one trial compared erlotinib plus docetaxel versus erlotinib or docetaxel alone [10], and one assessed erlotinib plus paclitaxel based chemotherapy versus paclitaxel based chemotherapy alone [16]. One trial did not show HRs for PFS or OS [22], but later it was showed in another article [12]. In three studies, the EGFR mutation status was confirmed in 601 patients [11, 13, 16]. One of these three studies reported data for subgroup analysis according to mutation status [11], one reported them in another article [23], and one did not provide enough data [13]. The characteristics of eligible studies are summarized in Table 1.

**Table 1:** Summary of Characteristics of the Included Studies. Abbreviations: E: erlotinib, Carb: carboplatin, Cisp: cisplatin, Pac: paclitaxel, Gem: Gemcitabine, Pem: Pemetrexed, NA: Not available

Study	Number of points	Dominant ethnicity	Female	Age (range)	Drug delivery	Treatment comparison	Non-smoker	EGFR-mutant	EGFR-wild-type
Herbst, 2005	1079	Caucasian/934	424	24–84	Continuous	E+Carb+Pac vs. Carb+Pac+Placebo	116	29	198
Gatzemeier, 2007	1159	Caucasian/1064	267	26–84	Continuous	E+Gem+Cisp vs. Gem+Cisp+Placebo	NA	NA	NA
Mok, 2009	154	Asian/145	46	27–79	Intercalated	E+Gem+Cisp or Carb vs. Gem+Cisp or Carb+Placebo	52	NA	NA
Thomas, 2013	146	NA	73	69–90	Continuous	E+Gem vs. E vs. Gem	240	24	19
Lee, 2013	240	Asian/240	157	NA	Intercalated	E+Pem vs. E vs. Pem	219	97	136
Wu, 2013	451	Asian/451	179	31–96	Intercalated	E+Gem+Cisp or Carb vs. Gem+Cisp or Carb+Placebo	219	97	136
Dittrich, 2014	165	Caucasian/157	64	31–84	Continuous	E+Pem vs. E vs Pem	24	NA	NA
Auliac, 2014	151	NA	115	NA	Intercalated	E+docetaxel vs. E vs. docetaxel	11	NA	98
Michael, 2014	54	Caucasian/49	22	38–86	Intercalated	E+Gem vs. Gem	8	NA	NA

### Risk of bias and publication bias assessment

Although all nine eligible trials reported that the participants were randomized into different treatment arms, three of them did not provide details about random sequence generation [12, 15, 16]. Only one trial showed concealment procedures [11]. Five trials were open-label, they did not mask either participants or personnel [8–10, 12, 13]. Five trials had independent persons who performed the outcome assessment [10, 11, 14–16], and one trial did not show details about the blinding of outcome assessment [12]. Six eligible trials conducted efficacy analysis on an intention-to-treat basis [8, 11, 13–16]; one trial missed two cases in both arms [10]; and one trial missed three patients who were still in treatment [9]. We believe that the outcomes were unlikely to have been affected in these instances. Six trials did not selectively report data [8–13], while the protocols of three trials were not available [14–16]. Therefore, we could not judge whether these three trials selectively reported data. No significant publication bias was detected for any of the measured outcomes by funnel plots.

### Progression free survival

**Fig 2:**
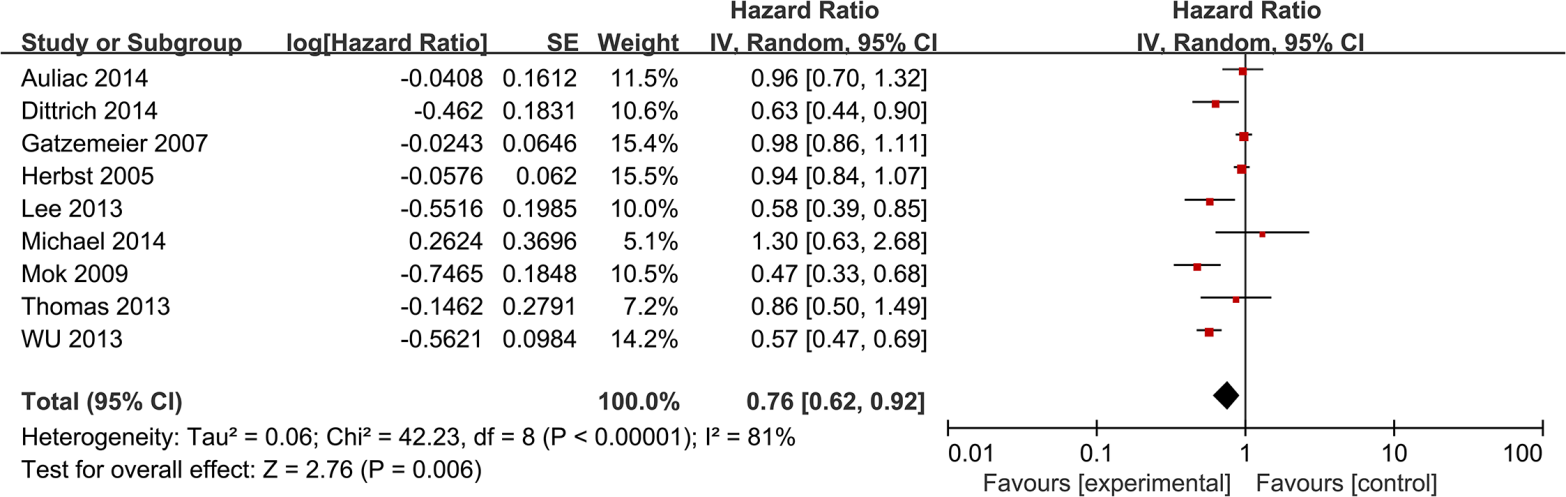
Forest Plot of Meta-analysis for PFS.

**Fig 3:**
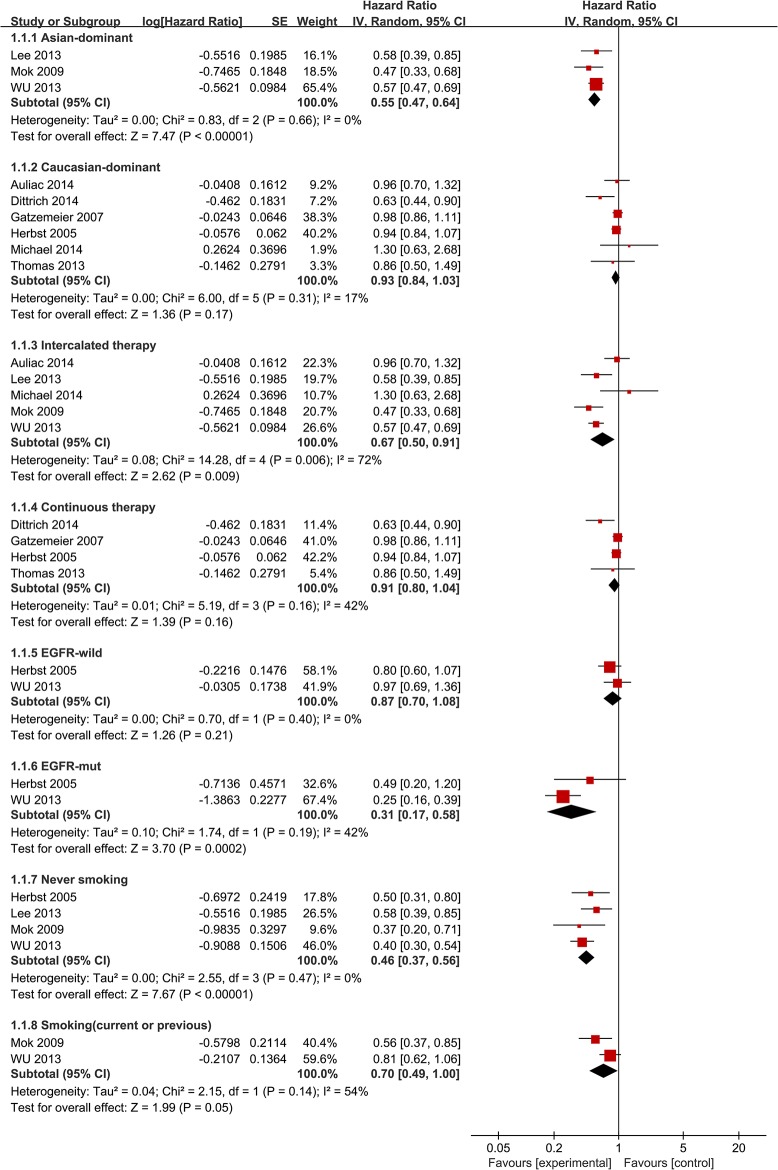
Forest Plot of Subgroup Analysis for PFS.

This meta-analysis showed a longer PFS in patients who received a combination of erlotinib and chemotherapy treatment (HR = 0.76 [95% CI 0.62, 0.92], P = 0.006) (Fig 2). The heterogeneity between studies was significant [χ2 = 14.28, df = 4 (P = 0.006); *I* ^*2*^ = 72%] (Fig 2). The pooled HR meta-analysis for intercalated erlotinib plus chemotherapy showed an improvement in PFS (HR = 0.67 [95% CI 0.50, 0.91], P = 0.009) (Fig 3). Meanwhile, continuous erlotinib plus chemotherapy treatment failed to show an improvement in PFS (HR = 0.91 [95% CI 0.80, 1.04], P = 0.16) (Fig 3). Subgroup analysis demonstrated improvements in PFS in never smoking patients (HR = 0.46 [95% CI 0.37, 0.56], P<0.00001) and patients with EGFR mutant tumors (HR = 0.31 [95% CI 0.17, 0.58], P = 0.0002) (Fig 3). No significant difference was shown in PFS between the chemotherapy plus erlotinib group and the chemotherapy group in patients with EGFR wild-type tumors (HR = 0.87 [95% CI 0.70, 1.08], P = 0.21) (Fig 3). In addition, smokers (current or previous) did not obtain a statistically significant benefit from erlotinib plus chemotherapy (HR = 0.70 [95% CI 0.49, 1.00], P = 0.05) (Fig 3).

### Overall survival

**Fig 4:**
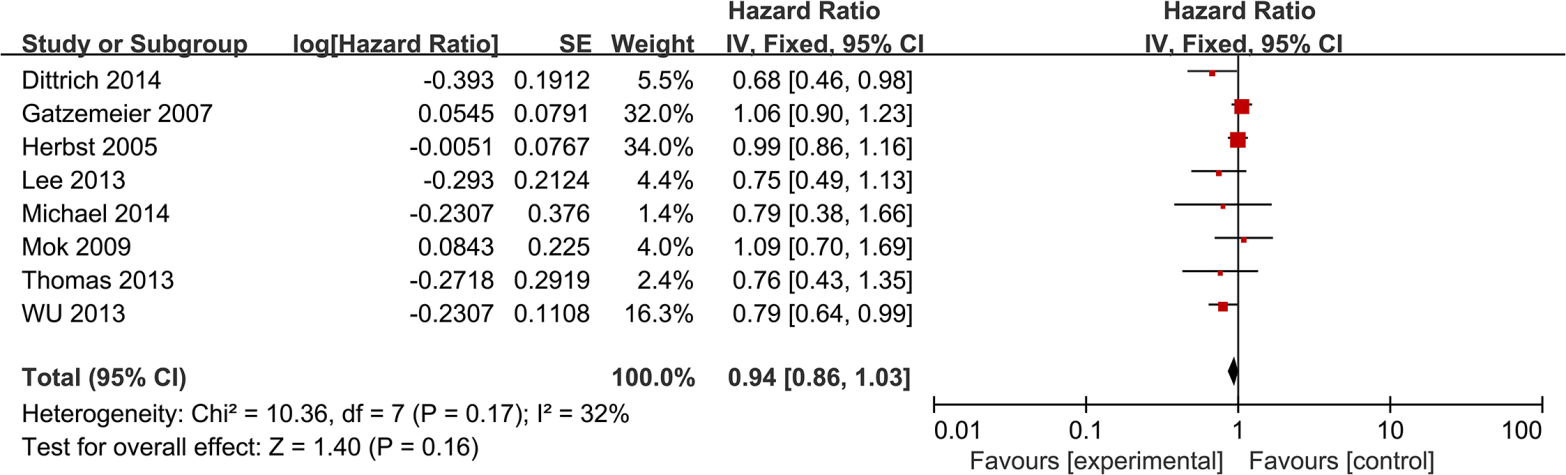
Forest Plot of Meta-analysis for OS.

**Fig 5:**
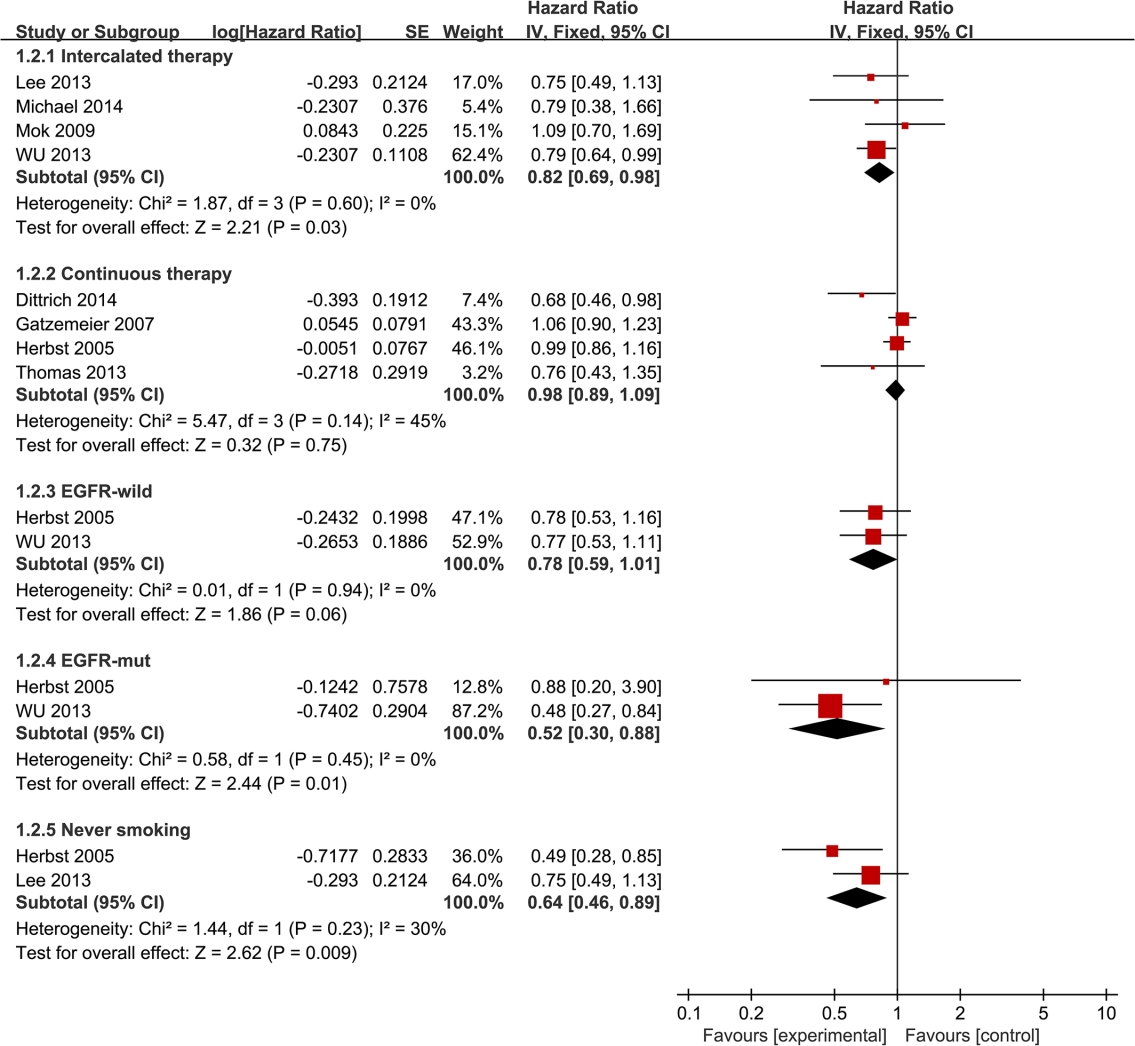
Forest Plot of Subgroup Analysis for OS.

HRs for OS data were available from 8 trials [8, 9, 11–16]. No statistically significant improvement was shown in OS (HR = 0.94 [95% CI 0.86, 1.03], P = 0.16) (Fig 4), and there was no significant heterogeneity [χ2 = 10.36, df = 7 (P = 0.17); *I* ^*2*^ = 32%]. Intercalated erlotinib plus chemotherapy treatment showed a modest but statistically significant improvement in OS (HR = 0.82 [95% CI 0.69, 0.98], P = 0.03) (Fig 5). Continuous erlotinib plus chemotherapy treatment failed to show an improvement in OS (HR = 0.98 [95% CI 0.89, 1.09], P = 0.75) (Fig 5). Subgroup analysis according to smoking status showed a statistically significant improvement in OS in never smoking patients (HR = 0.64 [95% CI 0.46, 0.89], P = 0.009) (Fig 5). Additionally, a statistically significant improvement in OS was observed in patients with EGFR mutant tumors (HR = 0.52 [95% CI 0.30, 0.88], P = 0.01) (Fig 5). No significant difference in OS was noted in patients with EGFR wild-type tumors (HR = 0.78 [95% CI 0.59, 1.01], P = 0.06) (Fig 5).

### Adverse events

Data for the grade 3 or 4 adverse events were available in five studies [9–11, 15, 16]. There were more incidences of grade 3 or 4 anemia (OR = 1.48 [95% CI 1.12, 1.97], P = 0.006), rash (OR = 12.34 [95% CI 5.65, 26.95], P<0.00001), and diarrhea (OR = 4.25 [95% CI 2.16, 8.38], P<0.0001) in the erlotinib and chemotherapy combination treatment. However, there was no difference in incidences of grade 3 or 4 neutropenia (OR = 1.02 [95% CI 0.83, 1.24]], P = 0.86), leucopoenia (OR = 1.31 [95% CI 0.80, 2.14], P = 0.29), or thrombocytopenia (OR = 1.26 [95% CI 0.91, 1.74], P = 0.17). Forest plots are shown in S1 Fig. The complete results are presented in S1 Table.

## Discussion

Over the past 20 years, chemotherapy has been the standard treatment for NSCLC. However, the survival benefit of chemotherapy is not significant. Many studies have attempted to improve the efficacy of chemotherapy by adding either another chemotherapeutic agent or a targeted agent to the treatment regimen. This meta-analysis of currently available data demonstrated that the combination of chemotherapy and erlotinib showed an improvement in PFS for advanced NSCLC. There was no evidence that the combination of chemotherapy and erlotinib improved OS when compared with chemotherapy alone. This is consistent with two previous systematic reviews, which also demonstrated that the addition of EGFR TKIs to chemotherapy showed an improvement in PFS, but not in OS [24, 25]. However, they did not analyze the efficacy of different schedules of erlotinib in combination with chemotherapy.

In subgroup analysis, intercalated erlotinib in combination with chemotherapy demonstrated improvements in both PFS and OS compared with chemotherapy alone. Previously, a randomized phase II study comparing intercalated erlotinib plus chemotherapy treatment to erlotinib alone also showed a significant improvement in OS [26]. In another randomized study, intercalated erlotinib plus pemetrexed showed 1.6-fold longer PFS compared with pemetrexed alone [17]. This intercalated therapy avoided the G1 arrest by erlotinib, thus optimizing the cell-cycle phase-dependent activity of chemotherapy. In other words, these data demonstrate that intercalated therapy is the most effective combinatorial strategy. This combinatorial strategy may benefit patients who have little benefit from EGFR-TKIs monotherapy [27]. In this meta-analysis, continuous erlotinib plus chemotherapy versus chemotherapy alone failed to show improvements in PFS and OS. A previous systematic review demonstrated that the addition of EGFR TKIs to platinum-based first-line chemotherapy did not significantly improve overall survival or time-to-disease progression [28]. The trials included in that systematic review all used continuous therapy strategy, which could partly explain the lack of benefit. This is also consistent with the theory that EGFR-TKIs cause G1 cell-cycle arrest, which inhibits the cell-cycle-dependent cytotoxic effects of chemotherapy. In this study, the trials included in the continuous therapy subgroup all dominantly enrolled Caucasian patients, which might be another reason for failure to show efficacy because previous studies had shown that Asian origin was a significant independent predictor for survival in EGFR-TKIs treatment [7]. Compared to Asian patients, Caucasian patients showed lower efficacy from oral EGFR-TKIs [29]. Similarly, this meta-analysis demonstrated that EGFR mutation was an important predictive biomarker for this treatment strategy. Among patients with EGFR mutant tumors, chemotherapy plus erlotinib demonstrated significant improvements in PFS (HR = 0.31 [95% CI 0.17, 0.58]) and OS (HR = 0.52 [95% CI 0.30, 0.88]). These data confirm the results of a previous phase II single arm clinical trial which showed that an intermittent schedule of erlotinib and gemcitabine based chemotherapy improved survival for patients with EGFR gene activating mutations [30]. Currently, several EGFR-TKIs such as erlotinib and gefitinib have been suggested as first-line treatments for patients with advanced EGFR mutation-positive NSCLC [31]. In this meta-analysis, no significant difference in PFS was found between the chemotherapy plus erlotinib group and the chemotherapy group in patients with EGFR wild-type tumors. Similarly, a previous single arm study failed to demonstrate an add-on effect of intermittent erlotinib with pemetrexed as a second-line treatment for patients with non-squamous NSCLC without EGFR mutations [32]. The association of chemotherapy plus erlotinib with improvement in PFS was significant in non-smokers, but it was not statistically significant in smokers. Previous studies showed that a history of not smoking was also a significant independent predictor for survival in EGFR-TKIs treatment [7].

The meta-analysis demonstrated a longer PFS in patients who received erlotinib plus chemotherapy, showing a high heterogeneity level [χ^*2*^ = 42.23, df = 8 (P = 0.006); *I* ^*2*^ = 81%] (Fig 2). To further explore this heterogeneity, we conducted subgroup analysis according to ethnicity. For PFS in Asian-dominant populations, HR = 0.55 [95% CI 0.47, 0.64] and the heterogeneity disappeared [χ^*2*^ = 0.83, df = 3 (P = 0.66); *I* ^*2*^ = 0%] (Fig 3). For PFS in Caucasian-dominant populations, HR = 0.93 [95% CI 0.84, 1.03] and the heterogeneity decreased [χ^*2*^ = 6, df = 5 (P = 0.31); *I* ^*2*^ = 17%] (Fig 3). Therefore, ethnicity could be the main reason for high heterogeneity. In subgroup analysis of intercalated combination of chemotherapy and erlotinib versus chemotherapy alone, there was also a high heterogeneity level [χ^*2*^ = 14.28, df = 4 (P = 0.006); *I* ^*2*^ = 72%]. When we excluded two studies that predominantly enrolled Caucasian populations, the heterogeneity disappeared [χ^*2*^ = 0.83, df = 2 (P = 0.66); *I* ^*2*^ = 0%]. Therefore, we believe that ethnicity is also the origin of this heterogeneity.

The present systematic review has some limitations. First, data could not be obtained from two included studies despite our contact with the primary investigators [17, 33]. Second, some trials such as TRIBUTE [16] enrolled 934 Caucasian patients and 154 others, while FASTACT [14] enrolled 145 Asian patients and 6 Caucasian patients. Due to limited data, we were unable to perform a pooled analysis according to ethnicity. To explore a high heterogeneity level in total PFS, we conducted subgroup analysis according to dominant ethnicity. What is more, given the probable bias due to the overlap of never smokers and EGFR mutants, data on never smokers are likely influenced by higher rate of EGFR mutants, since only 601 patients had EGFR status evaluated. We conclude that the combination of chemotherapy and erlotinib is a viable treatment option for patients with EGFR mutation. However, for patients with EGFR mutation-positive NSCLC, the current standard care is EGFR TKI alone. OPTIMAL study showed that compared with chemotherapy, erlotinib demonstrated a significant benefit in patients with advanced EGFR mutation-positive NSCLC, and median PFS was 13.1 months for erlotinib-treated patients versus 4.6 months for patients receiving chemotherapy [31]. In FASTACT-2, patients with EGFR mutation derived benefit from the combination treatment, and median PFS was 16.8 months [11]. We didn't address whether a combination treatment was better than erlotinib alone for patients with EGFR mutation-positive NSCLC. A head-to-head study is needed to answer this question. In this systematic review, we analyzed the efficacy of different schedules of erlotinib in combination with chemotherapy, and led to a conclusion that the intercalated schedule showed an improvement in PFS and OS, while the continuous schedule did not.

In conclusion, the combination of chemotherapy and erlotinib is a viable treatment option for patients with NSCLC, and intercalated administration is an effective combinatorial strategy. This treatment strategy could benefit patients who never smoked and patients with EGFR mutation-positive disease. As this treatment strategy was more toxic, it warrants further investigation.

## Supporting Information

S1 PRISMA Checklist PRISMA Checklist for this Review. (DOC)

S1 Fig Forest Plot of Meta-analysis for Grade 3/4 AEs. (TIF)

S1 Table Comparison of Grade 3/4 AEs between Erlotinib plus Chemotherapy and Chemotherapy Alone. (DOC)

S1 File A list of full-text excluded articles.doc. (DOC)
