# Supplementary figures and images for: Chemotherapy plus Erlotinib versus Chemotherapy Alone for Treating Advanced Non-Small Cell Lung Cancer: A Meta-Analysis
Source: PLoS One. 2015 Jul 6;10(7):e0131278. doi: 10.1371/journal.pone.0131278 (PMC4493135; doi:10.1371/journal.pone.0131278)

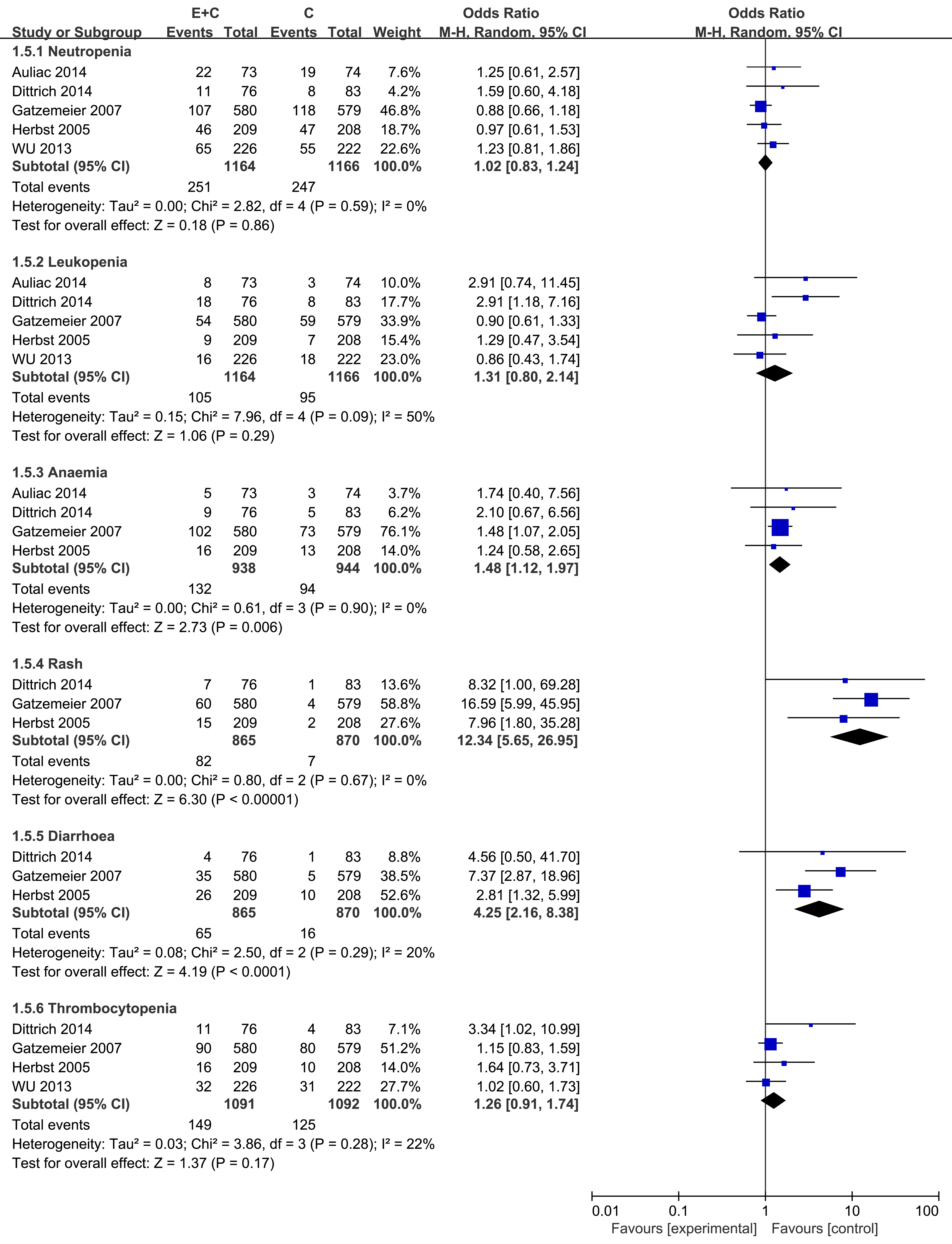

Supplement: S1 Fig — (TIF) [file pone.0131278.s002.tif]
