# Supplementary material for: Chemotherapy plus Erlotinib versus Chemotherapy Alone for Treating Advanced Non-Small Cell Lung Cancer: A Meta-Analysis
Source: PLoS One. 2015 Jul 6;10(7):e0131278. doi: 10.1371/journal.pone.0131278 (PMC4493135; doi:10.1371/journal.pone.0131278)
Supplement: S1 Table — (DOC) [file pone.0131278.s003.doc]

**S1 Table. Comparison of Grade 3/4 AEs between Erlotinib plus Chemotherapy and Chemotherapy Alone**

| CTCAE Grade 3/4 Toxicity | Trials | E+Chem | Chem | OR[95%CI] | P value | Heterogeneity I^2^ | |
| --- | --- | --- | --- | --- | --- | --- | --- |
|  |  |  |  |  |  | P value | I^2^ |
| Neutropenia | 5 | 251/1164 | 247/1166 | 1.02 [0.83, 1.24] | 0.86 | 0.59 | 0% |
| Anaemia | 4 | 132/938 | 94/944 | 1.48 [1.12, 1.97] | 0.006 | 0.90 | 0% |
| Leucopaenia | 5 | 105/1164 | 95/1166 | 1.31 [0.80, 2.14] | 0.29 | 0.09 | 50% |
| Rash | 3 | 82/865 | 7/870 | 12.34 [5.65, 26.95] | <0.00001 | 0.67 | 0% |
| Diarrhoea | 3 | 65/865 | 16/870 | 4.25 [2.16, 8.38] | <0.0001 | 0.29 | 20% |
| Thrombocytopenia | 4 | 149/1091 | 125/1092 | 1.26 [0.91, 1.74] | 0.17 | 0.28 | 22% |

Abbreviations: CTCAE = common terminology criteria for adverse events, AE = Adverse event, E: Erlotinib, Chem: Chemotherapy
