# Supplementary material for: Chemotherapy plus Erlotinib versus Chemotherapy Alone for Treating Advanced Non-Small Cell Lung Cancer: A Meta-Analysis
Source: PLoS One. 2015 Jul 6;10(7):e0131278. doi: 10.1371/journal.pone.0131278 (PMC4493135; doi:10.1371/journal.pone.0131278)
Supplement: S1 File — (DOC) [file pone.0131278.s004.doc]

A list of full-text excluded articles

Duplicate publication:

1. Eberhard DA, Johnson BE, Amler LC, Goddard AD, Heldens SL, Herbst RS, Ince WL, Jänne PA, Januario T, Johnson DH, Klein P, Miller VA, Ostland MA,Ramies DA, Sebisanovic D, Stinson JA, Zhang YR, Seshagiri S, Hillan KJ. Mutations in the epidermal growth factor receptor and in KRAS are predictive and prognostic indicators in patients with non-small-cell lung cancer treated with chemotherapy alone and in combination with erlotinib. Journal of Clinical Oncology:Epub 2005 Jul 25. 2005 Sep 1; 23(25): 5900-9.
2. Gatzemeier, U. Pluzanska, A. Szczesna, A. Kaukel, E. Roubec, J. Brennscheidt, U. Rosa, F. Mueller, B. Pawel, J. Results of a phase III trial of erlotinib (OSI-744) combined with cisplatin and gemcitabine (GC) chemotherapy in advanced non-small cell lung cancer (NSCLC) [abstract]. Journal of Clinical Oncology: ASCO annual meeting proceedings. Vol 22, No 14S (July 15 Supplement), 2004: 7010.
3. Herbst, R. S. Prager, D. Hermann, R. Miller, V. Fehrenbacher, L. Hoffman, P. Johnson, B. Sandler, A. B. Mass, R. Johnson, D. H. TRIBUTE - A phase III trial of erlotinib HCI (OSI-774) combined with carboplatin and paclitaxel (CP) chemotherapy in advanced non-small cell lung cancer (NSCLC) [abstract]. Journal of Clinical Oncology: ASCO annual meeting proceedings. September 1, 2005vol. 23 no. 25 5892-5899.
4. Miller, V. A. Herbst, R. Prager, D. Fehrenbacher, L. Hermann, R. Hoffman, P. Johnson, B. Sandler, A. B. Kris, M. G. Ramies, D. Long survival of never smoking non-small lung cancer (NSCLC) patients (pts) treated with erlotinib HCl (OSI-774) and chemotherapy: Sub-group analysis of TRIBUTE [abstract]. Journal of Clinical Oncology: ASCO annual meeting proceedings. Vol 22, No 14S (July 15 Supplement), 2004: 7061.
5. Tran, H. T. Zinner, R. Blumenschein, G. R. Oh, Y. W. Papadimitrakopoulou, V. A. Kim, E. S. Lu, C. Malik, M. Lum, B. Herbst, R. S. Pharmacokinetic study of the phase III, randomized, double-blind, multicenter trial of paclitaxel (Pac) and carboplatin (C) combined with erlotinib (E) or placebo in patients with advanced non-small cell lung cancer (NSCLC) [abstract]. Journal of Clinical Oncology: ASCO annual meeting proceedings. Vol 22, No 14S (July 15 Supplement), 2004: 2050.
6. Gandara, D. R. Yoneda, K. Shelton, D. Beckett, L. A. Ramies, D. A. Bloss, J. Herbst, R. S. Independent review of fatal interstitial lung disease (ILD) in TRIBUTE: paclitaxel + carboplatin {+/-} erlotinib in advanced non-small cell lung cancer (NSCLC). Journal of Clinical Oncology: ASCO annual meeting proceedings. Vol 24, No 18S (June 20 Supplement), 2006: 7071.
7. Yoneda, K. Y. Shelton, D. K. Beckett, L. A. Gandara, D. R. Independent review of interstitial lung disease associated with death in TRIBUTE (paclitaxel and carboplatin with or without concurrent erlotinib) in advanced non-small cell lung cancer. Journal of Thoracic Oncology. [J Thorac Oncol.](http://www.ncbi.nlm.nih.gov/pubmed/?term=Independent+review+of+interstitial+lung+disease+associated+with+death+in+TRIBUTE+(paclitaxel+and+carboplatin+with+or+without+concurrent+erlotinib)+in+advanced+non-small+cell+lung+cancer) 2007 Jun;2(6):537-43.
8. Lee, J. S. Ignacio, J. Yu, C. Zhou, C. Wu, Y. Chen, Y. Zhang, L. Jin, K. Johnston, M. Mok, T. S. FAST-ACT: A phase II randomized double-blind trial of sequential erlotinib and chemotherapy as first-line treatment in patients (pts) with stage IIIb/IV non-small cell lung cancer (NSCLC) [abstract no. 8031]. Journal of Clinical Oncology: ASCO annual meeting proceedings. Vol 26, No 15S (May 20 Supplement), 2008: 8031.
9. Stinchcombe, T. Bradford, D. S. Lee, C. B. Moore, D. T. Bakri, K. M. Taylor, M. A. Crane, J. M. Schwartz, G. Hensing, T. A. Socinski, M. A. Preliminary results of a randomized phase II trial of first-line treatment of gemcitabine (G) versus erlotinib (E) versus gemcitabine and erlotinib (GE) in patients 70 years or older with advanced non-small cell lung cancer (NSCLC). Journal of Clinical Oncology: 2010 ASCO Annual Meeting Abstracts. Vol 28, No 15_suppl (May 20 Supplement), 2010: 7576.
10. Lee, J. S. Wu, Y. L. Ladrera, G. Srimuninnimit, V. Sriuranpong, V. Thongprasert, S. Yu, C. J. Zhang, L. Margono, B. Mok, T. Intercalated erlotinib with gemcitabine/platinum in the first-line treatment of advanced non-small-cell lung cancer (NSCLC): The phase III, placebo-controlled fastact-II study. Journal of Thoracic Oncology 2011 6:6 SUPPL. 2 (S410-S411).
11. Stinchcombe, T. E. Peterman, A. H. Lee, C. B. Moore, D. T. Beaumont, J. L. Bradford, D. S. Bakri, K. Taylor, M. Crane, J. M. Schwartz, G. Hensing, T. A. McElroy Jr, E. Niell, H. B. Harper, H. D. Pal, S. Socinski, M. A. A randomized phase II trial of first-line treatment with gemcitabine, erlotinib, or gemcitabine and erlotinib in elderly patients (age ≥70 years) with stage IIIB/IV non-small cell lung cancer. Journal of Thoracic Oncology 2011 6:9 (1569-1577).
12. Tran, H. T. Zinner, R. G. Blumenschein Jr, G. R. Oh, Y. W. Papadimitrakopoulou, V. A. Kim, E. S. Lu, C. Malik, M. Lum, B. L. Herbst, R. S. Pharmacokinetic study of the phase III, randomized, double-blind, multicenter trial (TRIBUTE) of paclitaxel and carboplatin combined with erlotinib or placebo in patients with advanced Non-small Cell Lung Cancer (NSCLC). Invest New Drugs. 2011 Jun;29(3):499-505.
13. Michael, M. Pavlakis, N. Clingan, P. De Boer, R. Johnston, M. Clarke, S. A multi-centre randomized, open-label phase II trial of continuous erlotinib plus gemcitabine or gemcitabine as first-line therapy in ECOG PS2 patients with advanced non-small cell lung cancer. Oncol Rep. 2012 Sep;28(3):763-7.
14. Mok, T. Wu, Y. L. Thongprasert, S. Yu, C. J. Zhang, L. Ladrera, G. E. Srimuninnimit, V. Sriuranpong, V. Jennifer, S. T. Zhu, Y. Liao, M. Zhou, C. Pan, H. Lee, V. Chen, Y. M. Sun, Y. Margono, B. Jin, K. Truman, M. Lee, J. S. A randomized placebo-controlled phase III study of intercalated erlotinib with gemcitabine/platinum in first-line advanced non-small cell lung cancer (NSCLC): FASTACT-II. Journal of Clinical Oncology: 2012 ASCO Annual Meeting Abstracts. Vol 30, No 15_suppl (May 20 Supplement), 2012: 7519.
15. Stinchcombe, T. E. Roder, J. Grigorieva, J. Peterman, A. H. Lee, C. B. Moore, D. T. Socinski, M. A. A veristrat® analysis of samples from a randomized phase 2 trial of first-line therapy with gemcitabine, erlotinib, or gemcitabine and erlotinib in elderly patients (age≥ 70 years) with stage 3b/4 non-small cell lung cancer. Journal of Thoracic Oncology 2012 7:9 SUPPL. 4 (S209).
16. Von Pawel, J. Papai-Szekely, Z. Vinolas, N. Sederholm, C. Klima, M. Desaiah, D. Leschinger, M. Dittrich, C. A randomized phase 2 study of pemetrexed vs. pemetrexed+erlotinib in second-line treatment for locally advanced or metastatic, non-squamous NSCLC. Pneumologie 2012; 66 - P93.
17. Wu, Y. L. Liao, M. Zhu, Y. Zhou, C. Sun, Y. Pan, H. Wang, J. Qian, X. Mok, T. Truman, M. Zhang, L. Intercalated erlotinib with gemcitabine/platinum in the first-line treatment of chinese patients with advanced non-small-cell lung cancer (NSCLC): Subanalysis from the fastact-2 study. Journal of Thoracic Oncology 2012 7:11 SUPPL. 5 (S456).
18. Auliac, J. B. Greillier, L. Chouaid, C. Monnet, I. Le Caer, H. Falchero, L. Corre, R. Descourt, R. Bota, S. Berard, H. Schott, R. Bizieux-Thaminy, A. Fournel, P. Gervais, R. Dujon, C. Baize, N. Fraboulet, G. Paillotin, D. Abdiche, M. S. Locher, C. Marin, B. Vergnenegre, A. Randomized non comparative multicenter phase II study of sequential erlotinib with docetaxel versus docetaxel alone in patients with non small cell lung cancer (NSCLC) after failure of first line chemotherapy (tarseq): A GFPC 10.02 study. Journal of Thoracic Oncology 2013 8 SUPPL. 2 (S865).
19. Mok, T. Wu, Y. L. Lee, J. S. Yu, C. J. Sriuranpong, V. Wen, W. Tsai, J. Truman, M. Klughammer, B. Wu, L. Detection of EGFR-activating mutations from plasma DNA as a potent predictor of survival outcomes in FASTACT 2: A randomized phase III study on intercalated combination of erlotinib (E) and chemotherapy (C). Journal of Clinical Oncology, ASCO Annual Meeting Abstracts. Vol 31, No 15_suppl (May 20 Supplement), 2013: 8021.

Insufficient data:

1. Tianhong Li, Bilal Piperdi, William Vincent Walsh, Mimi Kim, Rasim Gucalp, Missak Haigentz, Venu Gopal Bathini, Xiaoxia Wu, Patricia Pasquinelli, Srikanth Gajavelli, Meera Sreedhara, Laurel A Beckett, Primo Lara, David R. Gandara, Roman Perez-Soler. Randomized phase II study of pharmacodynamic separation (PDS) of pemetrexed (Pem) and erlotinib (Erl) versus pem alone in patients (pts) with advanced non-small cell lung cancer (NSCLC). J Clin Oncol 31, 2013 (suppl; abstr 8097).
2. Boutsikou E, Kontakiotis T, Zarogoulidis P, Darwiche K, Eleptheriadou E, Porpodis K, et al. Docetaxel-carboplatin in combination with erlotinib and/or bevacizumab in patients with non-small cell lung cancer. Onco Targets Ther. 2013;6: 125.
